# Employment of Dentists in the Army

**Published:** 1897-05

**Authors:** Otto Arnold

**Affiliations:** Representative Ohio State Dental Society


### DENTISTS IN THE ARMY.

### Employment of Dentists in the Army.

*To the Dentists of the United States:*

At a recent meeting of the Ohio State Dental Society a resolution was adopted advocating the employment of dentists in the United States Army.

This resolution refers briefly to the necessity for dental service among the enlisted men, recommending that Congress be petitioned by dental societies to adopt such measures as will provide this special service. The resolution also provides for the appointment of a committee of one to represent the Ohio State Dental Society in this matter.

Our government expects its soldiers at all times to be able to render prompt and efficient service, and to this end demands primarily that the recruit be physically sound. To further this the government has established a standard of soundness which determines the fitness or unfitness of the applicant to serve in the army of his country.

Furthermore, our government, not content with an examination of the enlisted man, attempts to maintain the standard thus reached, and environs him with comfortable and sanitary quarters, furnishes him with sufficient and wholesome food, exacts adequate exercise and personal cleanliness; in short, throws about him every safeguard known to science for the furthering of his physical welfare. Furthermore, should he be stricken with disease, or disabled from any cause, the soldier is cared for in modern, well-equipped hospitals, and attended by skilled surgeons. Thus, every need is provided for. But is this absolutely true? How about his dental organs? Surely the soldier is but human, therefore, his teeth are an imporant part of his physique, and if lost or destroyed his whole system suffers more or less in consequence.

It seems not a little strange that our nation should not before this have included in its medical service the treatment of teeth.

Granted that the entrance examination be thorough and includes in its requirements the possession of a sound set of teeth, there will still remain the susceptibility to the insidious process of dental caries, which is no respecter of persons and has no limit; for diseases may come and go, bnt dental carries "goes on forever."

The mere act of passing from civil into military life does not render a man immune from dental carries, and it is well known that those thus afflicted suffer greatly, and, in fact, are often, from this cause totally disabled for a longer or shorter period from discharging their regular duties.

Therefore, it seems only reasonable that some adequate provision should be made for supplying this branch of medical science, thereby improving, and to that extent completing, the medical service of the army.

Thus far the only remedy at the soldier's command has been the extraction of the offending teeth—the operation usually performed by the hospital stewards, men wholly without dental training, and from whom it would be unreasonable to expect the necessary knowledge and skill for successfully practicing this branch of surgery.

Even if they were experts in tooth-extracting, the immediate result of their operations would be the loss of what were intended for useful organs, the certain impairment of the masticating faculties would ensue, together with an ultimate general effect upon the constitution of the patient.

Indifference to the needs of her servants is not in keeping with the generous policy which usually characterizes this nation; therefore, the dentists and medical men of this country, by virtue of their knowledge of existing conditions, earnestly appeal to every member of Congress to aid in the passage of a law that will supply this evident insufficiency.

Dentists, as members of the existing Examining Boards, would undoubtedly be valuable acquisitions in preventing the admission of men with defective teeth, in this particular alone rendering valuable service.

Furthermore, in cases of gunshot wounds and other injuries about the mouth and head, the dentist's special knowledge and skill in devising appliances necessary to obtain certain results would qualify him pre-eminently to manage the class of cases which the vicissitudes of war present.

Altogether considered the dentist is a necessary factor, no less in the army than in civil communities.

Dear reader, will you not personally lend a hand in this noble cause? Will you not enlist the services of your representative in Congress to support an enactment with this end in view, in the hope that they will speedily add this branch of healing to their otherwise perfect care of those who are the safeguard of our frontiers and coast line?

OTTO ARNOLD,
*Representative Ohio State Dental Society.*

